# PD‐L1 expression on tumor or stromal cells of nodal cytotoxic T‐cell lymphoma: A clinicopathological study of 50 cases

**DOI:** 10.1111/pin.12950

**Published:** 2020-05-18

**Authors:** Daisuke Yamashita, Kazuyuki Shimada, Kei Kohno, Yasunori Kogure, Keisuke Kataoka, Taishi Takahara, Yuka Suzuki, Akira Satou, Ayako Sakakibara, Shigeo Nakamura, Naoko Asano, Seiichi Kato

**Affiliations:** ^1^ Department of Pathology and Laboratory Medicine Nagoya University Hospital Aichi Japan; ^2^ Department of Pathology Kobe City Hospital Organization Kobe City Medical Center General Hospital Hyōgo Japan; ^3^ Department of Hematology and Oncology Nagoya University Graduate School of Medicine Aichi Japan; ^4^ Division of Molecular Oncology National Cancer Center Research Institute Tokyo Japan; ^5^ Department of Surgical Pathology Aichi Medical University Hospital Aichi Japan; ^6^ Department of Molecular Diagnostics Nagano Prefectural Suzaka Hospital Nagano Japan; ^7^ Department of Pathology and Molecular Diagnostics Aichi Cancer Center Hospital Aichi Japan

**Keywords:** cytotoxic molecule, Epstein–Barr virus, neoplastic PD‐L1 expression, peripheral T‐cell lymphoma‐not otherwise specified (PTCL‐NOS), TCR phenotype

## Abstract

Inhibitors of programmed cell‐death 1 (PD‐1) and programmed cell‐death ligand 1 (PD‐L1) have revolutionized cancer therapy. Nodal cytotoxic T‐cell lymphoma (CTL) is characterized by a poorer prognosis compared to nodal non‐CTLs. Here we investigated PD‐L1 expression in 50 nodal CTL patients, with and without EBV association (25 of each). We identified seven patients (14%) with neoplastic PD‐L1 (nPD‐L1) expression on tumor cells, including three males and four females, with a median age of 66 years. One of the seven cases was TCRαβ type, three were TCRγδ type and three were TCR‐silent type. Six of the seven cases exhibited a lethal clinical course despite multi‐agent chemotherapy, of whom four patients died within one year of diagnosis. Morphological findings were uniform, with six cases showing centroblastoid appearance. Among nPD‐L1^+^ cases, two of three examined had structural variations of *PD‐L1* disrupting 3′‐UTR region. Notably, all of the TCRγδ‐type nodal CTL cases showed nPD‐L1 or miPD‐L1 positivity (3 and 10 cases, respectively). TCRγδ‐type cases comprised 42% of nPD‐L1^+^ cases (*P* = 0.043 *vs*. PD‐L1^−^), and 35% of miPD‐L1^+^ cases (*P* = 0.037 *vs*. PD‐L1^−^). The results indicate that PD‐L1^+^ nodal CTL cases, especially of the TCRγδ type, are potential candidates for anti‐PD‐1/PD‐L1 therapies.

**Abbreviations**
AITL: angioimmunoblastic T‐cell lymphoma
ALCL: anaplastic large cell lymphoma
ALK: anaplastic lymphoma kinase
CHOP: cyclophosphamide, hydroxydaunorubicin, vincristine (Oncovin), and prednisolone
CTLs: cytotoxic T‐cell lymphomas
DLBCL: diffuse large B‐cell lymphoma
EBV: Epstein–Barr virus
ENKTL: extranodal NK/T‐cell lymphomas
FFPE: formalin, and embedded in paraffin
HPF: high‐power field
miPD‐L1: microenvironmental PD‐L1
NK: natural killer
nPD‐L1: neoplastic PD‐L1
PD‐1: programmed cell‐death 1
PD‐L1: programmed cell‐death ligand 1
PIT: Prognostic Index for PTCL
PTCL‐NOS: peripheral T‐cell lymphoma‐not otherwise specified
TCR: T‐cell receptor

## 1 INTRODUCTION

Over the past two decades, we have gradually elucidated the clinicopathological spectrum and biological behaviors of primary nodal cytotoxic T‐cell lymphoma (CTL) with and without Epstein–Barr virus (EBV) association.^1^, ^2^, ^3^, ^4^, ^5^, ^6^, ^7^, ^8^, ^9^, ^10^, ^11^, ^12^, ^13^, ^14^, ^15^ This disease typically exhibits a diffuse monomorphic pattern of proliferation of large cells, often having a centroblastoid appearance. It more commonly occurs in elderly patients or in the setting of immunodeficiency. In the 2017 WHO classification, primary nodal CTL is described as a variant of peripheral T‐cell lymphoma (PTCL), not otherwise specified (NOS);^16^ however, additional data may lead to its designation as a separate entity. Nodal CTLs are frequently regarded as aggressive neoplasms due to their resistance to multi‐agent anthracycline‐based chemotherapy.^17^

Cancer therapy has been revolutionized by inhibitors of programmed cell‐death 1 (PD‐1) and programmed cell‐death ligand 1 (PD‐L1). Indeed, Kwong *et al*. reported the strong effectiveness of PD‐1 blockade in relapsed or refractory cases of natural killer (NK)/T‐cell lymphoma^18^ with confirmed tumor cell expression of neoplastic PD‐L1 (nPD‐L1). Since NK/T‐cell lymphoma is considered a representative cytotoxic neoplasm, these results suggest that anti‐PD‐1/PD‐L1 therapies may also be effective against nodal CTL with nPD‐L1 expression.

In our present study, we report the clinicopathological features of nodal CTL cases showing positive nPD‐L1 expression or positive PD‐L1 expression on non‐malignant microenvironment immune cells (miPD‐L1), with particular focus on T‐cell receptor (TCR) γδ type.

## 2 MATERIALS AND METHODS

### 2.1 Patients

This study enrolled patients who were diagnosed with PTCL‐NOS by lymph node biopsy according to the 2017 WHO classification,^16^ between January 1982 and April 2019. Inclusion criteria were absence of B‐cell markers, and positivity for at least one T‐cell antigen (CD3, CD4, CD5, CD8 or CD45RO) based on immunohistochemistry or flow cytometry. All included patients exhibited positive expression of at least one cytotoxic molecule. EBV presence was evaluated using *in situ* hybridization of EBV‐encoded small nuclear early region, with a cut‐off of >50% positivity among neoplastic cells. Patients were also clinically evaluated for nodal disease. Our analysis excluded patients with lymphoepithelioid (Lennert) lymphoma, angioimmunoblastic T‐cell lymphoma (AITL), anaplastic lymphoma kinase (ALK)‐positive or ALK‐negative anaplastic large cell lymphoma (ALCL), ATLL, primary cutaneous T‐cell lymphoma or NK/T‐cell lymphoma of the nasal type.

We identified a total of 50 evaluable cases of nodal CTL with paraffin blocks available for analyses, including 41 from our previous study.^14^ Our study protocol was approved by the institutional review board of Nagoya University (No. 1066‐3).

### 2.2 Histopathology

Tissue samples were fixed in 10% formalin, and embedded in paraffin (FFPE). The cases were reviewed by three pathologists (DY, SK and SN), and were divided into four morphological groups based on cell nuclei shape: centroblastoid, pleomorphic, mixed and unspecified. The centroblastoid group included cases where >50% of neoplastic cells were large and had oval‐to‐round vesicular nuclei with fine chromatin, morphologically resembling diffuse large B‐cell lymphoma (DLBCL) cells. The pleomorphic group included cases where over two‐thirds of tumor cells exhibited pleomorphic features with irregular nuclei folding. The mixed group included tumors comprising a mixture of medium and large cells. Despite the varying cell size, mixed morphology tumors exhibited lower cellular atypia than tumors with pleomorphic morphology. Finally, the unspecified group comprised cases with biopsy specimens too small to achieve a good consensus regarding morphology. Cells were also evaluated for presence of elongated nuclei.

### 2.3 Immunophenotypic and ISH analysis

**Figure 1:**
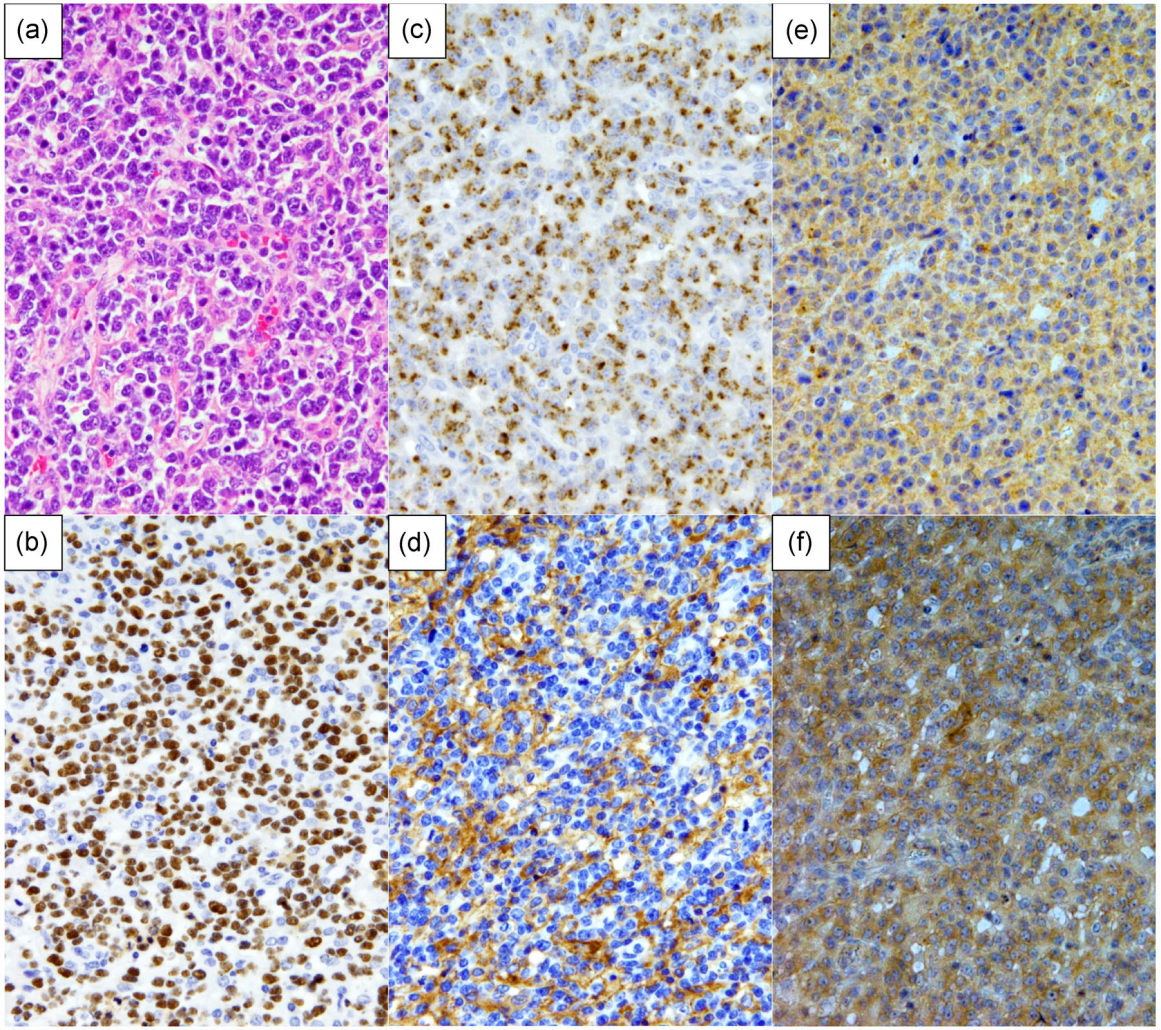
Light microscopy images of nodal EBV^+^ CTL samples from Case 1 (Tables 1 and 2). Nuclear morphology was examined by hematoxylin and eosin staining, revealing centroblastoid morphology (**a**). Other samples were immunostained for EBV‐encoded small RNA (EBER) (**b**), TIA‐1 (**c**), PD‐L1 (clone SP142) (**d**), TCRγ (**e**) and TCRδ (**f**). Original magnification: 400×.

The FFPE sections were subjected to immunoperoxidase analysis with the following monoclonal antibodies: CD4, CD5 and CD56 (Novocastra Laboratories, Newcastle, UK); CD3, CD8, L26/CD20, Ber‐H2/CD30 and ALK1 (Dako, Santa Clara, CA, USA); βF1 (TCR β chain; T Cell Science, Cambridge, MA, USA); TCR 1153 (TCR‐γ; clone γ 3.20) and TCRδ constant region (clone 5A6.E9; Thermo Fisher Scientific, Waltham, MA, USA)^13^; TIA‐1 (Coulter Immunology, Hialeah, FL); granzyme B (Monosan, Uden, the Netherlands); PD‐L1 (clone SP142 from Spring Bioscience, Pleasanton, CA, USA; 28‐8 from Abcam plc, Cambridge, UK; and E1J2J from Cell Signaling Technology, Danvers, MA, USA) and ALK 5A4.^19^ Reactions were considered positive with a cut‐off of 30%. Positive nPD‐L1 expression (Fig. 1) was defined by a cut‐off of ≥10% of tumor cells, as in our previous report.^14^ The nPD‐L1‐negative CTL cases were divided into two subpopulations based on whether they exhibited PD‐L1 positivity on non‐malignant microenvironment immune cells. Microenvironmental PD‐L1 (miPD‐L1)‐positive CTL cases were defined as cases with ≥10 PD‐L1‐positive non‐malignant microenvironment immune cells per high‐power field (HPF), as previously reported.^20^, ^21^

To evaluate the presence of EBV small ribonucleic acids, we subjected formalin‐fixed, paraffin‐embedded sections to *in situ* hybridization using EBV‐encoded small nuclear early region (EBER) oligonucleotides, as previously reported.^22^

The FFPE tissue sections were also used for dual‐color FISH analysis using a SPEC CD274, PDCD1LG2/CEN9 Dual Color Probe (Zytovision, Bremerhaven, Germany).

### 2.4 Detection of *PD‐L1* genetic alterations

Detection of *PD‐L1* genetic alterations was performed as previously described.^23^ Briefly, structural variations (SVs) affecting *PD‐L1* were explored using targeted‐capture sequencing with a custom SureSelect library (Agilent Technologies, Santa Clara, CA, USA), which can capture the entire sequence of the *PD‐L1* gene, including their exons, introns and 5′‐ and 3′‐UTRs. Sequencing data were obtained using the Illumina NextSeq500 platform with a standard 150‐bp paired‐end read protocol.^23^ SVs were detected using the Genomon pipeline (https://github.com/Genomon‐Project) as previously described with modification. Putative SVs were manually curated and further filtered by removing those (i) with both breakpoints are out of bait region; (ii) with both breakpoints are within an immunoglobulin/T‐cell receptor region; (iii) with <6 supporting reads in tumor; (iv) with allele frequency in tumor <0.02; (v) with sum of Max_Over_Hang <200 bp; or (vi) present in any of unmatched normal samples. SV breakpoints were visually inspected using IGV.

### 2.5 Statistical analysis

We evaluated correlations between the two groups using Fisher's exact test and Student's *t* test. Patient survival data were analyzed using the Kaplan‐Meier method and log‐rank test. Our analysis excluded survivors with a follow‐up period of less than 6 months. Univariate and multivariate analyses were performed using a Cox proportional hazard regression model. All statistical analyses were performed using the graphical user interface for R, EZR (The R Foundation for Statistical Computing, Vienna, Austria).^24^

## 3 RESULTS

### 3.1 Clinicopathological characteristics

**Figure 2:**
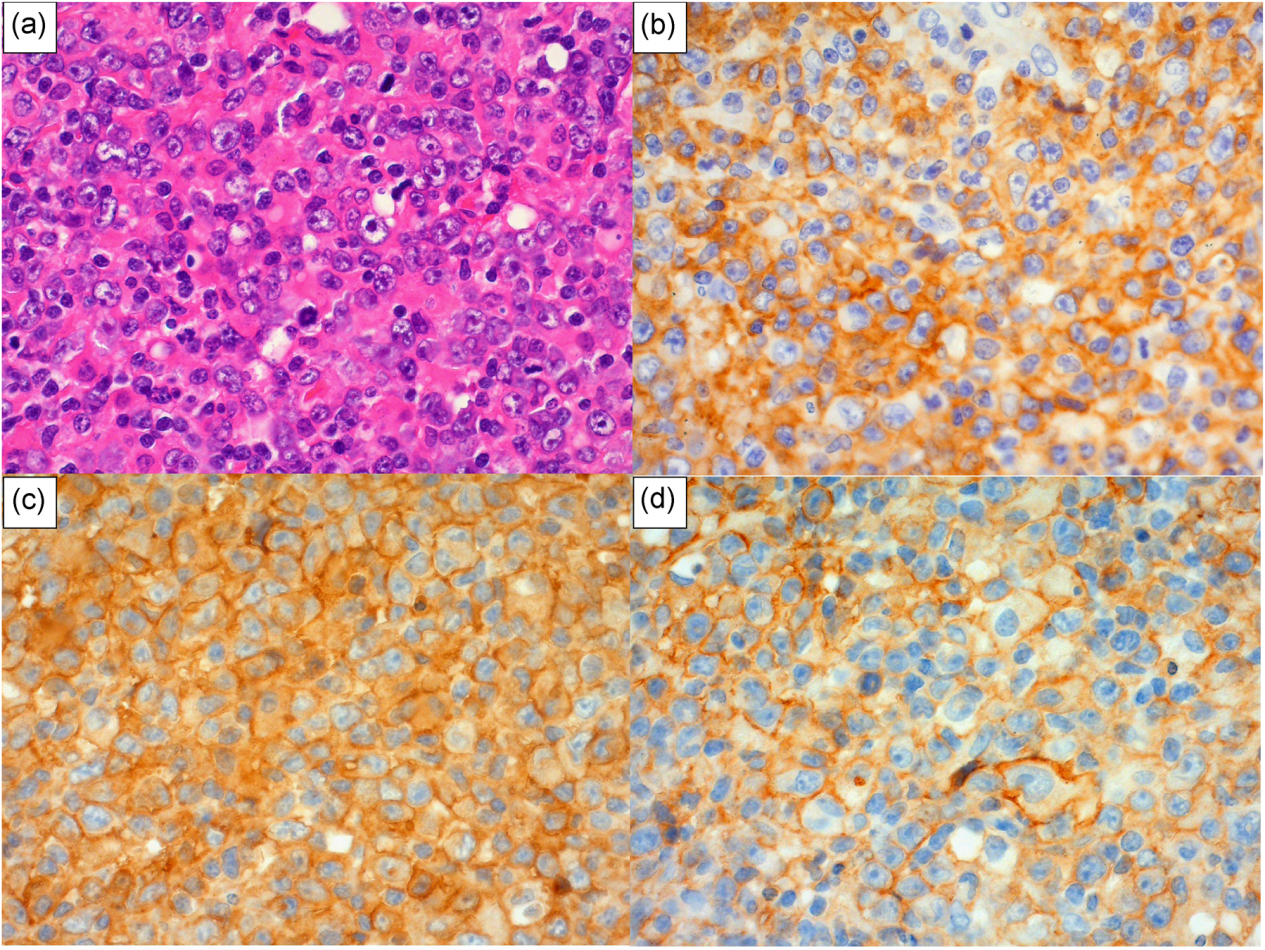
Light microscopy images of nodal EBV^−^ CTL samples from Case 6 (Tables 1 and 2). Nuclear morphology was examined by hematoxylin and eosin staining, revealing centroblastoid morphology (**a**). Other samples were immunostained for PD‐L1, showing positive staining with the anti‐PD‐L1 antibodies of clones SP142 (**b**), 28‐8 (**c**) and E1J2J (**d**). Original magnification: 400×.

**Figure 3:**
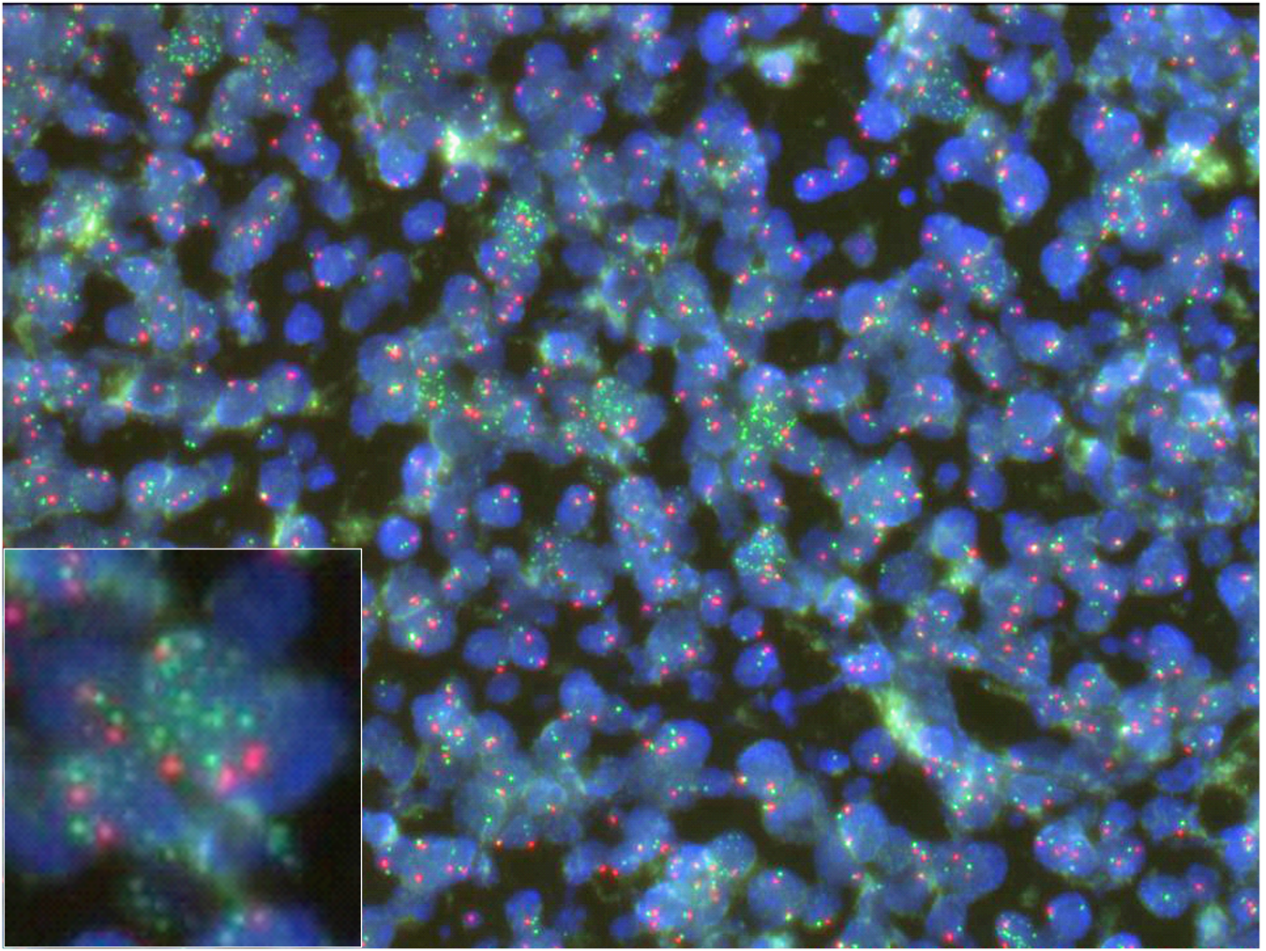
Fluorescent *in situ* hybridization (FISH) analysis of the programmed death‐ligand 1 (PD‐L1) gene amplification in Case 7, using a SPEC CD274/PD‐L1 (green signal), PDCD1LG2/CEN9 (red signal) Dual Color Probe. Original magnification: 400×. Inset: enlarged view of tumor cells.

We divided our cohort of 50 patients with nodal CTL into three subgroups based on immunohistochemical PD‐L1 positivity (clone SP142). Seven patients (14%) had nPD‐L1‐positive nodal CTL (nPD‐L1^+^), 31 patients (62%) had nPD‐L1^−^ nodal CTL with positive PD‐L1 expression in non‐malignant microenvironment immune cells (miPD‐L1^+^) and 12 (24%) lacked both neoplastic and microenvironment PD‐L1 expression (PD‐L1^−^). Tables 1 and 2 summarize the clinicopathological features of the seven nPD‐L1^+^ CTL cases in the current series. Of these patients, four were female, and the age range was 32–76 years (median, 66 years). All patients were Japanese, and their presenting symptoms included fever and weakness. At presentation, all patients exhibited lymphadenopathy, and anemia was present in all examined patients, with hemoglobin ranging from 7.4 to 11.9 g/dL. Four patients had a decreased platelet count at presentation, usually markedly so, with a range of 15–39 × 10^9^/L. This thrombocytopenia appeared to correlate with prognostic index for PTCL (PIT) group 3 or 4. Three patients exhibited hemophagocytosis. Extranodal involvement was documented in the liver in two patients, and in the gastrointestinal tract in one patient. Bone marrow positivity was not detected throughout the entire clinical course. Two patients (Cases 1 and 4) had undergone prior immunosuppressive drug therapy due to idiopathic thrombocytopenic purpura (Case 1) or chronic active Epstein–Barr virus infection (Case 4). Of the seven patients, six received systemic multi‐agent chemotherapy, while the remaining male patient exhibited a rapidly lethal clinical course within one week. Five patients exhibited progressive disease following therapy, and died at 2 weeks to 21 months after diagnosis. Only one patient achieved complete remission with CHOP (cyclophosphamide, hydroxydaunorubicin, vincristine (Oncovin) and prednisolone), and underwent autologous stem cell transplantation. She currently remains in complete remission at 18.2 months following transplantation. All cases exhibited high‐grade morphology comprising a monomorphic population of large transformed cells, with predominantly centroblastoid appearance in six cases, and pleomorphic appearance in one case. The tumor cells were of medium‐to‐large size, and contained a moderate amount of pale eosinophilic cytoplasm. The cytoplasmic borders were indistinct. In the cases with centroblastoid appearance, the nuclei were round, vesicular or slightly irregular in shape, with moderately dispersed chromatin and small distinct nucleoli. In the case with a pleomorphic appearance, the nuclei were highly indented or lobulated. In all seven cases, tumor cells were positive for TIA‐1 and PD‐L1 (clone SP142). The other immunophenotypes varied. Of seven tested cases, five were CD3^+^, two CD4^+^, three CD5^+^, one CD8^+^, one CD56^+^ and four granzyme B^+^. Three of four tested cases were CD30^+^, and two of three tested cases were perforin 1^+^. All cases exhibited nPD‐L1 positivity in ≥20% of lymphoma cells (range, 20–100%). Five of the seven evaluable cases were miPD‐L1‐negative. Three cases (Cases 5, 6 and 7) were additionally immunostained with the anti‐PD‐L1 antibodies of clone 28‐8 and E1J2J, yielding positive staining of tumor cells in all examined samples (Fig. 2). Of the seven cases, one was TCRαβ type, three were TCRγδ type and three were TCR‐silent type. Four of the seven cases harbored EBV on the nuclei according to EBER *in situ* hybridization. Using the conventional ALK1 antibody, ALK expression was not detected in our series. The CD274/PD‐L1 gene copy number status was assessed in three cases (Case 1, 6 and 7), in which FFPE sections were available for FISH analysis. Gene amplification was detected in only one (Case 7; Fig. 3), but not the other two. Targeted‐capture sequencing was performed to detect SVs involving *PD‐L1* in these three cases (Case 1, 6 and 7), which revealed two of the cases were with disrupted *PD‐L1* 3′‐UTR (Fig. S1). Case 6 had a tandem duplication of *PD‐L1* involving the entire coding sequence. Of note, Case 7 had multiple inter‐ and intra‐chromosomal rearrangements involving *PD‐L1* and other regions of five different chromosomes, suggesting the presence of ‘chromothripsis’ (Table S1).

**Table 1:** Clinical features of the seven nPD‐L1^+^ CTL cases

Case	Age/Sex	PS >1	Stage	B symptoms present	Extranodal site ≥ 2 sites	Hemophagocytosis	IPI high‐intermediate/high
1	76/M	+	IV	+	−	NA	+
2	60/F	−	III	+	+	−	+
3	32/F	−	II	−	−	NA	−
4	40/F	−	IV	+	−	+	−
5	66/M	+	III	+	−	+	+
6	72/F	+	II	+	−	+	+
7	73/M	−	II	−	−	NA	−

PIT group 3/4	Hemoglobin[g/dL]	Platelets (×10^9^/L)	Prior immunosuppressive drug therapy	Chemotherapty	ASCT	Response rate	Follow‐up (mo)
+	11.2	38	+	No therapy			Dead (0.2)
−	7.4	233	−	COP	Without	PR	Dead (4.5)
−	NA	NA	NA	CHOP	With	CR	Alive (27)
−	10	39	+	CHOP	With	CR	Dead (21)
+	8.6	29	NA	CHOP	Without	NR	Dead (2.2)
+	8.5	15	−	THP‐COP	Without	NR	Dead (0.4)
+	11.9	230	−	CHOP	Without	PR	Dead (6.5)

Abbreviations: ASCT, autologous stem cell transplantation; CHOP, cyclophosphamide doxorubicin vincristine and predonisone; CTL, cytotoxic molecule(CM)‐positive peripheral T‐cell lymphoma; CR, complete response; F, female; IPI, International Prognostic Index; M, male; NA, not available; NR, no response; PIT, prognostic index for PTCL; PR, partial response; PS, performance status; TCR, T‐cell receptor; THP‐COP, pirarubicin cyclophosphamide vincristine predonisolone.

**Table 2:** Pathological features of the seven nPD‐L1^+^ CTL cases

Case	nPD‐L1 (%)	EBER	CD3	CD4	CD5	CD8	CD30	CD56	TIA‐1	Granzyme B	Perforin 1	TCR phenotype	Morphology
1	20	+	+	−	−	+	NA	−	+	+	+	γδ	Centroblastoid
2	30	−	+	−	+	−	+	−	+	−	+	silent	Centroblastoid
3	70	−	+	+	−	−	+	+	+	+	NA	silent	Centroblastoid
4	70	+	+	+	+	−	NA	−	+	+	NA	αβ	Centroblastoid
5	80	+	+	−	−	−	NA	−	+	+	NA	γδ	Pleomorphic
6	80	−	−	−	−	−	+	−	+	−	−	silent	Centroblastoid
7	100	+	+	−	+	−	−	−	+	−	NA	γδ	Centroblastoid

Abbreviations: CTL indicates cytotoxic molecule (CM)‐positive peripheral T‐cell lymphoma; PD‐L1, programmed cell‐death ligand 1; TCR, T‐cell receptor.

The PD‐L1^−^ subgroup included no TCRγδ‐type cases. In contrast, TCRγδ‐type cases comprised 42% of nPD‐L1^+^ cases (*P* = 0.043 *vs*. PD‐L1^−^; Table 3), and 35% of miPD‐L1^+^ cases (*P* = 0.037 *vs*. PD‐L1^−^). Among our 50 cases, 13 (26%) were categorized as TCRγδ type, including three nPD‐L1^+^ and 10 miPD‐L1^+^ cases. All 13 patients with TCRγδ type showed positive nPD‐L1 or miPD‐L1 expression. Compared to the miPD‐L1^+^ subgroup, cases of nPD‐L1^+^ nodal CTL less frequently had advanced clinical stages (*P* = 0.040), but more frequently showed anemia (*P* = 0.018).

**Table 3:** Clinicopathological characteristics of 50 patients with nodal CTL

	nPD‐L1 positve nodal CTL (*n* = 7) (*n* (%))	miPD‐L1 positve nodal CTL (*n* = 31) (*n* (%))	PD‐L1 negative nodal CTL (*n* = 12) (*n* (%))	*n versus* others *P*	*n versus mi P*	*n versus* negative *P*	*mi versus* negative *P*
Age at diagnosis (median (range)) (years)	66 (32–76)	65 (21–81)	69 (29–78)	0.58	0.61	0.55	0.75
Age at diagnosis > 60 years	4/7 (57)	20/31 (64)	8/12 (66)	0.69	1	1	1
Sex (male/female)	3/4	18/13	8/4	0.43	0.68	0.38	0.74
PS >1	3/7 (42)	15/26 (57)	7/11 (63)	0.44	0.67	0.63	1
Clinical Stage III/IV	4/7 (57)	27/29 (93)	10/12 (83)	0.053	0.040	0.31	0.57
B symptoms present	5/7 (71)	18/25 (72)	7/12 (58)	1	1	0.66	0.47
Extranodal site ≥ 2 sites	1/7 (14)	8/29 (27)	2/12 (16)	1	0.65	1	0.70
Extranodal sites							
Bone marrow	0/7 (0)	9/28 (32)	2/12 (16)	0.18	0.15	0.51	0.45
Liver	2/6 (33)	8/27 (29)	3/12 (25)	1	1	1	1
Skin and/or soft tissue	0/7 (0)	2/29 (6)	1/12 (8)	1	1	1	1
GI tract	1/7 (14)	0/29 (0)	1/12 (8)	0.27	0.19	1	0.29
Hemophagocytosis	3/4 (75)	8/27 (29)	2/11 (18)	0.081	0.12	0.077	0.69
IPI_high‐intermediate/high	4/7 (57)	24/29 (82)	7/11 (63)	0.35	0.17	1	0.23
PIT group 3/4	4/7 (57)	25/29 (86)	7/11 (63)	0.33	0.12	1	0.18
Hb <13 g/dL (male) or Hb <11 g/dL (female)	6/6 (100)	11/27 (40)	6/11 (54)	0.022	0.018	0.10	0.49
Platelets <130 ×10^9^/L	4/6 (66)	15/28 (53)	6/11 (54)	0.68	0.67	1	1
Serum LDH > normal	6/7 (85)	26/29 (89)	8/12 (66)	1	1	0.60	0.17
CRP > normal	4/4 (100)	20/23 (86)	6/7 (85)	1	1	1	1
Prior immunosuppressive drug therapy	2/5 (40)	4/22 (18)	1/8 (12)	0.26	0.30	0.51	1
History of autoimmune disease	1/5 (20)	4/24 (16)	1/9 (11)	1	1	1	1
Treatment							
No therapy	1/7 (14)	4/29 (13)	1/12 (8)	1	1	1	1
CT with anthracycline	5/7 (71)	21/29 (72)	9/11 (81)	1	1	1	0.70
CT without anthracycline	1/7 (14)	4/29 (13)	0/11 (0)	0.53	1	0.38	0.56
ASCT	2/7 (28)	3/29 (10)	4/12 (33)	0.60	0.24	1	0.17
Response							
CR	2/6 (33)	7/21 (33)	3/11 (27)	1	1	1	1
PR	2/6 (33)	4/21 (19)	1/11 (9)	0.30	0.59	0.52	0.64
NR	2/6 (33)	10/21 (47)	7/11 (63)	0.66	0.66	0.34	0.47
Morphology							
Centroblastoid	6/7 (85)	16/31 (51)	4/12 (33)	0.10	0.20	0.057	0.33
Pleomorphic	1/7 (14)	9/31 (29)	6/12 (50)	0.41	0.65	0.17	0.29
Mixed	0/7 (0)	5/31 (16)	2/12 (16)	0.573	0.56	0.51	1
Unspecified	0/7 (0)	1/31 (3)	0/12 (0)	1	1	1	1
Immunophenotype							
TIA‐1	7/7 (100)	27/30 (90)	10/12 (83)	1	1	0.51	0.61
Granzyme B	4/7 (57)	23/28 (82)	10/11 (90)	0.12	0.31	0.25	0.66
cyCD3	6/7 (85)	28/29 (96)	11/12 (91)	0.38	0.36	1	0.51
CD4	2/7 (28)	9/29 (31)	5/12 (41)	1	1	0.66	0.72
CD5	3/7 (42)	12/31 (38)	5/12 (41)	1	1	1	1
CD8	1/7 (14)	14/29 (48)	7/12 (58)	0.11	0.20	0.15	0.73
CD30	3/4 (75)	9/22 (40)	6/8 (75)	0.60	0.31	1	0.22
CD56	1/7 (14)	6/30 (20)	2/12 (16)	1	1	1	1
EBER	4/7 (57)	16/31 (51)	5/12 (41)	1	1	0.65	0.74
TCR phenotype							
αβ	1/7 (14)	4/28 (14)	7/11 (63)	0.66	1	0.066	0.004
γδ	3/7 (42)	10/28 (35)	0/11 (0)	0.39	1	0.043	0.037
TCR‐silent	3/7 (42)	9/28 (32)	2/11 (18)	0.66	0.67	0.33	0.46
NK‐cell type	0/7 (0)	5/28 (17)	2/11 (18)	0.57	0.56	0.50	1
Indolent nodal CTL	0/7 (0)	3/31 (9)	3/12 (25)	0.58	1	0.26	0.33

Abbreviations: ASCT, autologous stem cell transplantation; CTL, cytotoxic molecule(CM)‐positive peripheral T‐cell lymphoma; CR, complete remission; CRP, C‐reactive protein; CT, chemotherapy; cyCD3, cytoplasmic CD3; EBER, EBV‐encoded small RNA; GI tract, gastrointestinal tract; Hb, hemoglobin; IPI, International Prognostic Index; LDH, lactate dehydrogenase; miPD‐L1, microenvironmental PD‐L1; NK, natural killer; NR, no response; nPD‐L1, neoplastic PD‐L1; PD‐L1, programmed cell‐death ligand 1; PIT, prognostic index for PTCL; PR, partial remission; PS, performance status; TCR, T‐cell receptor; Indolent nodal CTL means αβ or NK‐cell type of TCR phenotype in nodal EBV‐negative and CD5‐positive CTL.

**Figure 4:**
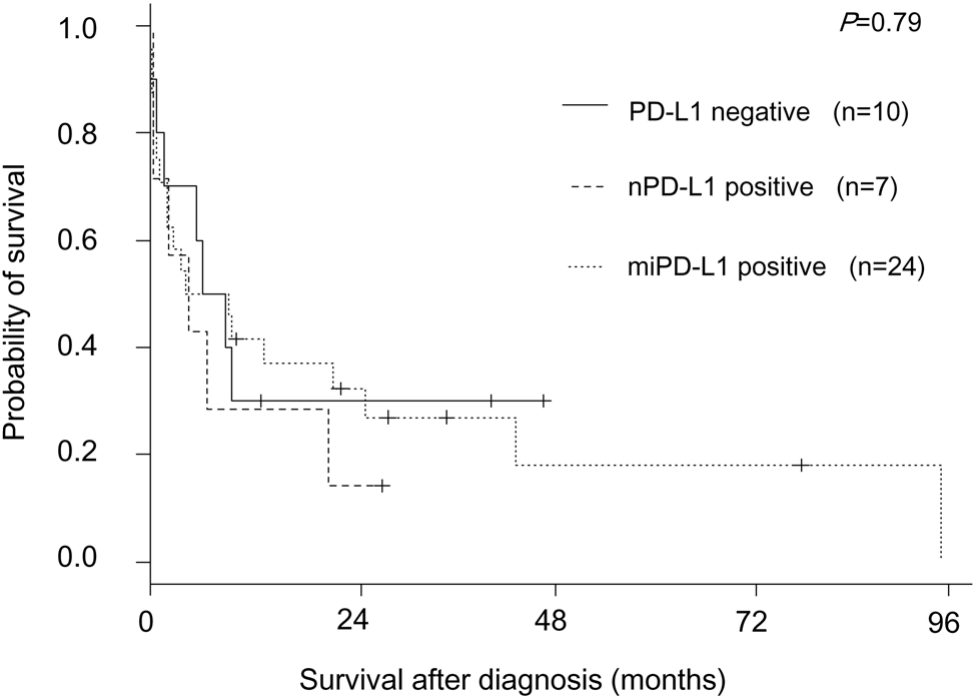
Survival curves for nodal CTL patients from the neoplastic PD‐L1‐positive (nPD‐L1^+^), microenvironmental PD‐L1‐positive (miPD‐L1^+^) and PD‐L1^−^ groups.

The present series included six cases with indolent CD5^+^ cytotoxic nodal T‐ or NK‐cell lymphoproliferative disease affecting patients of ≤60 years old,^14^ none of which exhibited neoplastic expression of PD‐L1. Despite the presence of this prognostically favorable patient subset in the nPD‐L1‐negative groups, the overall survival curves overlapped among the nPD‐L1^+^, miPD‐L1^+^ and PD‐L1^−^ patient groups (Fig. 4).

## 4 DISCUSSION

The current 2017 WHO classification of lymphoid neoplasms includes the severe distinct disease entities cytotoxic T‐ and NK‐cell lymphomas, which are both characterized by constant expression of cytotoxic molecules, as well as frequent EBV association, CD8 positivity, TCRγδ phenotype, and predilection for certain anatomical sites.^16^ These features are distinct from non‐cytotoxic T‐cell lymphomas, which are exemplified by angioimmunoblastic T‐cell lymphoma, adult T‐cell leukemia/lymphoma, mycosis fungoides, *etc*.^17^ They generally share an aggressive clinical course due to resistance to ordinal multi‐agent chemotherapy, although several unique indolent cytotoxic diseases have been identified over the last decade, including primary cutaneous acral CD8^+^ T‐cell lymphoma,^5^ lymphomatoid gastropathy,^25^ NK‐cell enteropathy^26^ and an enteropathy‐like indolent NK‐cell proliferation of the female genital tract.^27^ We recently elucidated an indolent CD5^+^ cytotoxic nodal T‐ or NK‐cell lymphoproliferative disease affecting patients of ≤60 years old in our Japanese series of nodal CTL cases.^14^ In that investigation, we simultaneously identified five cases of nPD‐L1‐positive nodal overt CTL, but without corresponding clinicopathological findings.

Neoplastic PD‐L1 expression has been thoroughly analyzed in ALK^+^ ALCL, which is activated by multiple oncogenic signaling pathways downstream of ALK activity.^28^, ^29^, ^30^ Indeed, in our series, most ALK^+^ ALCL cases (19 of 20; 95%) were positive for nPD‐L1 (clone SP142) (data not shown), which is in contrast to the minimal nPD‐L1 expression observed in ALK‐negative ALCL.^31^ On the other hand, Kwong *et al*. reported that a humanized anti‐PD‐1 monoclonal antibody was highly effective against relapsed/refractory extranodal NK/T‐cell lymphomas (ENKTL).^18^, ^32^ Using gene expression profiling, Ng *et al*. found higher *PD‐L1* expression on tumor and non‐tumor cells in nodal EBV^+^ CTL cases than in ENKTL, suggesting potential therapeutic implications for anti‐PD‐1 treatment in nodal EBV^+^ CTL.^33^ Kataoka *et al*. also recently reported PD‐L1‐related somatic aberrations in 23% of ENKTL cases, 57% of aggressive NK‐cell leukemia cases and 17% of systemic EBV‐positive T‐cell lymphoproliferative disorders.^34^ However, only limited data are presently available regarding nPD‐L1 expression in the other cytotoxic lymphomas of the T‐ or NK‐cell lineage.

In our present study, we identified seven cases of nodal CTL with nPD‐L1 expression, which constituted a small subset (14%) of the present series. Their overall clinicopathological features were generally in line with those previously documented in this aggressive disease.^1^, ^2^, ^3^, ^4^, ^5^, ^6^, ^7^, ^8^, ^9^, ^10^, ^11^, ^12^, ^13^, ^14^, ^15^, ^35^ Of these seven cases, four (57%) were associated with EBV, one was TCRαβ type, three were TCRγδ type and three were TCR‐silent type. According to the study definition, all presented with lymphadenopathy, but were generally in more localized stages compared with other cases lacking nPD‐L1 expression. Six cases followed a lethal clinical course despite multi‐agent chemotherapy, with death within one year of diagnosis in four cases. Prognosis did not significantly differ (*P* = 0.71; Fig. S2) between the groups delineated based on PD‐L1 positivity on tumor and non‐malignant microenvironment immune cells, based on the presently used cut‐off values (*e.g*., 40% of the latter). Hypothetically, this result might be biased due to the aggressive clinical course of many nodal CTL cases. Indeed, among nodal CTL patients, we have failed to identify biological prognostic indicators, except for TCRγδ phenotype among EBV^+^ patients^13^ and CD5 positivity^14^ in EBV^−^ patients. No definite conclusions can be drawn due to the paucity of enrolled cases. Thus, future studies are needed to clarify the prognostic or predictive significance of nPD‐L1 in patients with nodal CTL.

Notably, all patients with nodal CTL of the TCRγδ type showed positive nPD‐L1 or miPD‐L1 expression. PD‐L1 expression on neoplastic cells and on infiltrating immune cells appears to have a major effect on response to immunotherapy.^36^, ^37^, ^38^ Our present data showed that cases of nodal TCRγδ‐type CTL frequently exhibited positive PD‐L1 expression on tumor cells and on non‐malignant microenvironment immune cells. Lymphocytes of the γδ T‐cell lineage are immunologically characterized by potent cytotoxicity and interferon‐γ (IFN‐γ) production, which may be related to PD‐L1.^39^, ^40^ PD‐L1 can be induced on tumor cells and on stromal immune cells in response to IFNγ.^41^, ^42^ Although it is presently difficult to explain the apparent link between PD‐L1 expression and the TCRγδ phenotype of the tumor cells, future clinical trials should explore whether anti‐PD‐1/PD‐L1 therapies may improve our therapeutic approach for these aggressive diseases. We previously demonstrated that TCRγδ type had a negative prognostic impact in nodal EBV‐positive CTL and in gastrointestinal T‐cell lymphoma,^13^, ^43^ and here we have elucidated its possible predicative aspect regarding PD‐L1 positivity.

In this study, we investigated SVs involving *PD‐L1* gene of three out of seven nPD‐L1^+^ cases. Among them, two of three (66%) had SVs of *CD274/PD‐L1* disrupting 3′‐UTR region, which are reported to stabilize *PD‐L1* transcripts, thereby upregulating PD‐L1 expression.^44^ Case 6 was shown to have tandem duplication involving the entire coding sequence of *CD274/PD‐L1* gene. Case 7 had complex inter‐ and intra‐chromosomal events involving *CD274/PD‐L1* gene, which might be the underling mechanism of *CD274/PD‐L1* gene amplification detected by FISH. In Case 7, the gene translocations did not affect the coding sequence of *CD274/PD‐L1* and C terminus of *PD‐L1* was intact, therefore, the upregulated PD‐L1 expression was detectable by SP142 antibody. Although the number of the cases was too small to draw any definite conclusions, our data suggest that various genomic aberrations involving *CD274/PD‐L1* gene cause upregulations of nPD‐L1 expression (clone SP142) in nodal CTL cases.

Interestingly, our CTL cases with nPD‐L1 expression (clone SP142) shared uniform morphological findings, with a centroblastoid appearance in six cases and a pleomorphic appearance in one case, beyond their diversity regarding EBV association and TCR phenotypes. The centroblastoid morphological appearance was first documented in nodal CTL cases by our group in 1999,^2^ and often poses a problem in the differential diagnosis from DLBCL in the routine practice of pathologists. Since this initial report, we have continuously highlighted its importance in characterizing the clinicopathological distinctiveness of nodal CTL *versus* extranodal NK/T‐cell lymphoma, as the latter entity rarely displays a centroblastoid appearance.^22^ This close association between nPD‐L1 expression and centroblastoid appearance may provide additional support to our assertion of the clinicopathological distinctiveness of nodal CTL compared to NK/T‐cell lymphoma and nodal PTCL‐NOS of the non‐cytotoxic type.

In conclusion, here we assessed PD‐L1 expression in 50 patients with nodal CTL, and revealed neoplastic PD‐L1 expression in a small subset (14%). Notably, all TCRγδ‐type cases showed nPD‐L1 or miPD‐L1 positivity. nPD‐L1 or miPD‐L1 expression in cases of nodal CTL may still provide a strong rationale for the use of PD‐L1 blockade (*e.g*., anti‐PD‐1/PD‐L1 therapy) as a potential treatment for these patients, due to the frequency of a rapidly fatal outcome.

## DISCLOSURE STATEMENT

None declared.

## AUTHOR CONTRIBUTIONS

DY collected data, analyzed data, interpreted data and wrote the manuscript. SK conceived the research project, collected data, analyzed data, interpreted data, wrote the manuscript and gave final approval. KS, KK, AS, YS, TT, AS, SN and NA collected data, analyzed data, interpreted data, and critically reviewed the manuscript. All authors approved the final version of the manuscript.

## Supporting information

Additional Supporting Information may be found in the online version of this article at the publisher's website.

**Figure S1** Targeted‐capture sequencing in three cases (Case 1, 6, and 7) revealed that Case 6 and Case 7 had structural variation of *CD274/PD‐L1* gene. Summary of genetic aberrations involving *CD274/PD‐L1* gene in these two cases. Type of alterations is indicated by color.

**Figure S2** Survival curves for nodal CTL patients of the neoplastic PD‐L1‐positive (nPD‐L1^+^), microenvironmental PD‐L1‐positive (miPD‐L1^+^), and PD‐L1^−^ groups. Groups were determined based on the PD‐L1 positivity of examined tumor and non‐malignant microenvironment immune cells, with a cut‐off of 40% of the latter.

**Table S1** PD‐L1 genetic alterations detected by targeted‐capture sequencing. Case 7 had multiple inter‐ and intra‐chromosomal rearrangements involving PD‐L1 and other regions of five different chromosomes.

Supporting information.
