# Supplementary figures and images for: PD‐L1 expression on tumor or stromal cells of nodal cytotoxic T‐cell lymphoma: A clinicopathological study of 50 cases
Source: Pathol Int. 2020 May 18;70(8):513–22. doi: 10.1111/pin.12950 (PMC7496983; doi:10.1111/pin.12950)

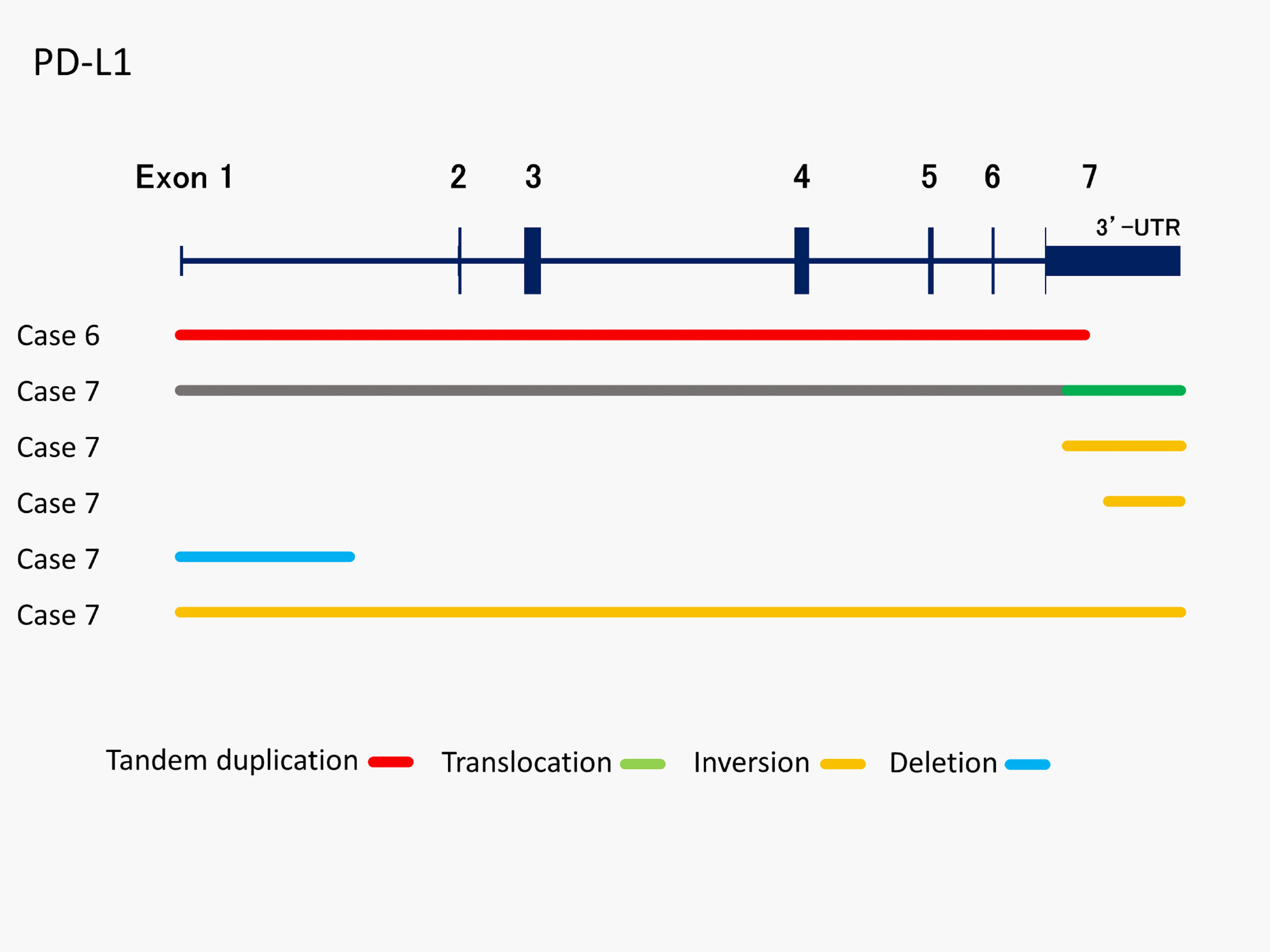

Supplement: Supplementary file 1 — Figure S1 Targeted‐capture sequencing in three cases (Case 1, 6, and 7) revealed that Case 6 and Case 7 had structural variation of CD274/PD‐L1 gene. Summary of genetic aberrations involving CD274/PD‐L1 gene in these two cases. Type of alterations is indicated by color. [file PIN-70-513-s001.jpg]

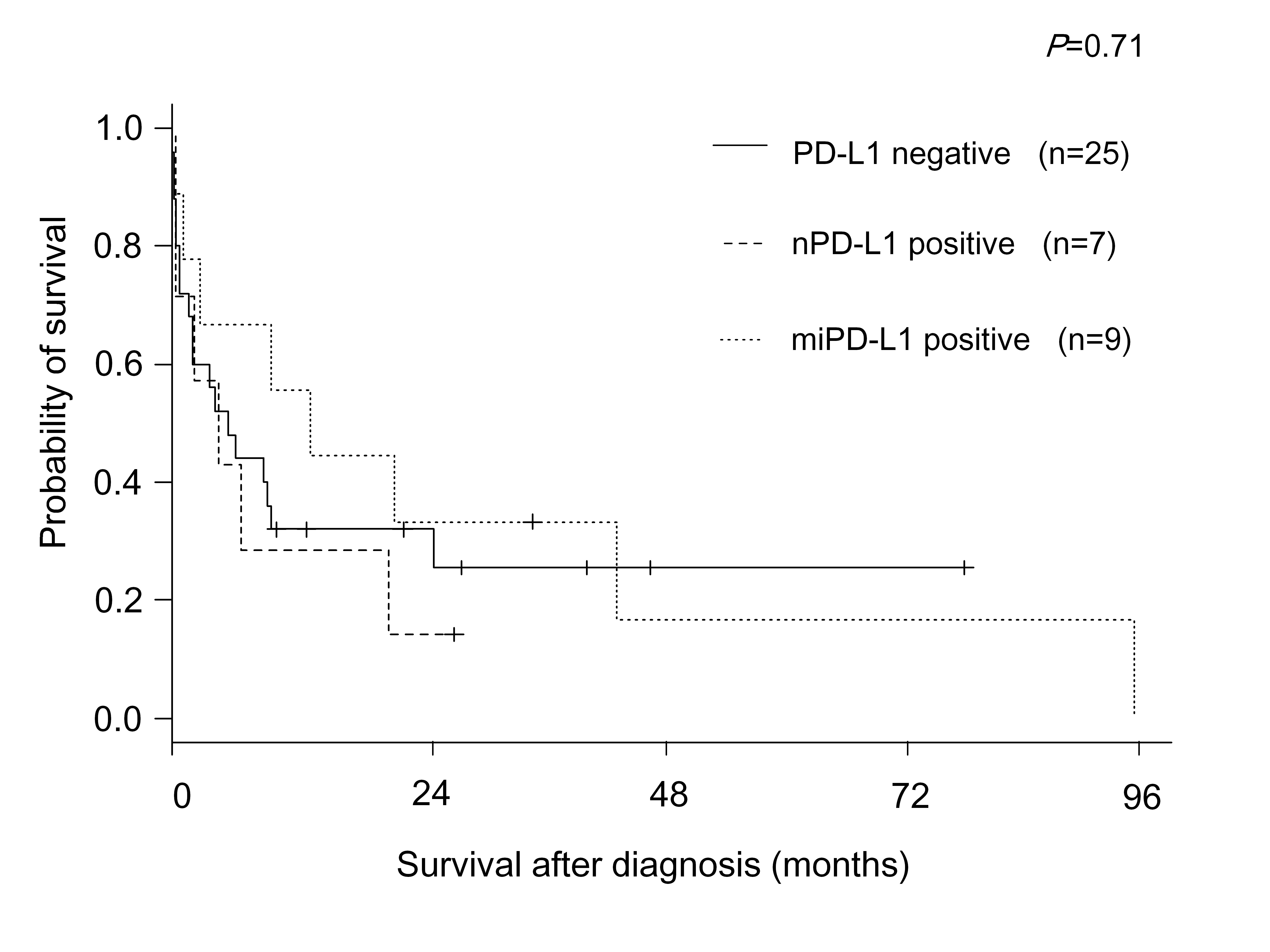

Supplement: Supplementary file 2 — Figure S2 Survival curves for nodal CTL patients of the neoplastic PD‐L1‐positive (nPD‐L1+), microenvironmental PD‐L1‐positive (miPD‐L1+), and PD‐L1− groups. Groups were determined based on the PD‐L1 positivity of examined tumor and non‐malignant microenvironment immune cells, with a cut‐off of 40% of the latter. [file PIN-70-513-s002.jpg]
