# Supplementary material for: PD‐L1 expression on tumor or stromal cells of nodal cytotoxic T‐cell lymphoma: A clinicopathological study of 50 cases
Source: Pathol Int. 2020 May 18;70(8):513–22. doi: 10.1111/pin.12950 (PMC7496983; doi:10.1111/pin.12950)
Supplement: Supplementary file 4 — Supporting information. [file PIN-70-513-s004.docx]

**SUPPORTING INFORMATION**

**Figure S1** Targeted-capture sequencing in 3 cases (Case 1, 6, and 7) revealed that Case 6 and Case 7 had structural variation of *CD274/PD-L1* gene. Summary of genetic aberrations involving *CD274/PD-L1* gene in these 2 cases. Type of alterations is indicated by color.

**Figure S2** Survival curves for nodal CTL patients of the neoplastic PD-L1-positive (nPD-L1^+^), microenvironmental PD-L1-positive (miPD-L1^+^), and PD-L1^−^ groups. Groups were determined based on the PD-L1 positivity of examined tumor and non-malignant microenvironment immune cells, with a cut-off of 40% of the latter.

**Supplemental Table S1** PD-L1 genetic alterations detected by targeted-capture sequencing. Case 7 had multiple inter- and intra-chromosomal rearrangements involving PD-L1 and other regions of 5 different chromosomes.
